# Patient‐derived tumor organoids highlight the potential of precision medicine in managing pancreatic ductal adenocarcinoma

**DOI:** 10.1002/ijc.35443

**Published:** 2025-04-28

**Authors:** Christine Nitschke, Charline Phan, Yara Souto, Philipp Walter, Mara Goetz, Gediminas Simkus, Jacob Thastrup, Ronald Simon, Jürgen Kupper, Jakob Izbicki, Steven A. Johnsen, Thilo Hackert, Marianne Sinn, Harriet Wikman, Faik G. Uzunoglu, Tabea M. Sturmheit

**Affiliations:** ^1^ Department of General, Visceral and Thoracic Surgery University Medical Center Hamburg‐Eppendorf Hamburg Germany; ^2^ Mildred Scheel Cancer Career Center Hamburg Hamburg Germany; ^3^ 2cureX Copenhagen Denmark; ^4^ Robert Bosch Center for Tumor Diseases Stuttgart Germany; ^5^ Institute of Tumor Biology University Medical Center Hamburg‐Eppendorf Hamburg Germany; ^6^ Institute of Pathology University Medical Center Hamburg‐Eppendorf Hamburg Germany; ^7^ II. Medical Clinic (Department of Oncology) University Medical Center Hamburg‐Eppendorf Hamburg Germany

**Keywords:** drug testing, pancreatic cancer, precision medicine, tumor organoids

## Abstract

Pancreatic ductal adenocarcinoma (PDAC) ranks among the most lethal cancers, with only 20% of patients qualifying for curative treatment at diagnosis. Three‐dimensional tumor organoids capturing patient‐specific features of PDAC serve as a valuable disease model. We employed this technology to assess drug sensitivities of patient‐derived tumor organoids to clinically relevant drugs and combinations, evaluated culture success rates, and correlated in vitro data with clinicopathological and follow‐up information. Tumor organoid cultures were established from PDAC patients undergoing surgical resection (or liver biopsy) and follow‐up at a single medical center. Patient‐derived cultures displaying sustained growth were analyzed regarding their molecular subtype and utilized for functional drug sensitivity testing (f‐DST). Correlative analyses of our PDAC patient cohort (*n* = 67; *n* = 42 patients with curative tumor resection and *n* = 25 palliative patients) revealed a link between tumor organoid growth and reduced patient survival. Furthermore, drug sensitivity profiles (obtained of 10 patient‐derived cultures) revealed notable inter‐individual differences and mirrored clinical responses to administered drug therapies. f‐DST was applicable across tumor organoid cultures of both classical and basal subtype, according to the Purity Independent Subtyping of Tumors (PurIST) classifier. This pilot study confirms the feasibility of deriving and maintaining tumor organoid cultures from heterogeneous samples. Cultures displaying sustained proliferation correlated positively with advanced‐stage tumors (Tumour, Node, Metastasis (UICC) stages III and IV). Individual patient case analyses integrating in vitro drug sensitivity profiles with clinical follow‐up data suggest that f‐DST using tumor organoids could guide future therapeutic strategies. In summary, tumor organoids offer insights into patient‐specific responses to treatment, highlighting the potential of precision medicine in managing this challenging cancer.

## Graphical abstract

What's new?

Only about one‐fifth of patients with pancreatic ductal adenocarcinoma (PDAC) qualify for curative treatment approaches employing adjuvant chemotherapy. Tools to predict PDAC response to chemotherapeutic regimens, however, are lacking. Here, using patient‐derived PDAC tumor organoids, the authors evaluated specific responses to drug combinations commonly administered in the adjuvant setting. Drug sensitivity analyses identified variability in tumor responses, with good overlap between in vitro testing data and actual clinical responses of matched patients. Tumor organoid growth further was associated with reduced patient survival. The findings highlight the utility of tumor organoids for providing insight into patient‐specific responses to chemotherapeutic regimens for PDAC.

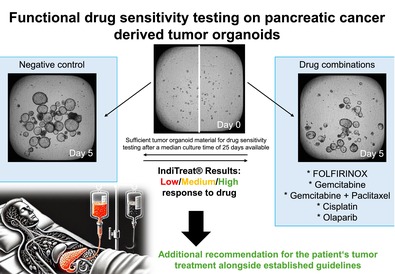

**Abbreviations**
3D: three‐dimensional
BSA: bovine serum albumin
cDNA: complementary DNA
CI: confidence interval
CT: computed tomography
EMT: epithelial–mesenchymal transition
f‐DST: functional drug sensitivity testing
GFR: growth factor reduced
HR: hazard ratio
OS: overall survival
PBS: phosphate‐buffered saline
PDAC: pancreatic ductal adenocarcinoma
PFS: progression‐free survival
qPCR: real‐time quantitative PCR
RFS: recurrence‐free survival
RNA: ribonucleic acid

## 1 INTRODUCTION

Pancreatic ductal adenocarcinoma (PDAC) is one of the deadliest cancers due to late diagnosis, unspecific symptoms, high metastatic potential, and poorly efficient therapies.^1^, ^2^ At diagnosis, only a minority of patients qualify for a curative treatment approach.^3^ The gold standard for resectable PDAC is a multimodal approach comprising radical surgery and adjuvant FOLFIRINOX chemotherapy, a therapy with 5‐fluorouracil (5‐FU), leucovorin, irinotecan, and oxaliplatin.^4^ Neoadjuvant chemotherapy (with modified FOLFIRINOX) is increasingly employed for advanced PDAC to achieve a technically resectable tumor anatomy.^4^ In palliative treatment, chemotherapy is administered to extend progression‐free survival (PFS) and to improve quality of life.^5^

At present, there are no tools to predict a patient's response to chemotherapeutic regimens.^6^ The only broadly applied tool to assess tumor responses during and after chemotherapy is imaging‐based (computed tomography [CT]). In PDAC, this technology has limitations due to the specific peritumoral stromal reaction.^7^

PDAC is a heterogeneous disease with a variety of histological and molecular subtypes. One can distinguish between two clinically most relevant subtypes: First, the classical subtype (~80%), rich in adhesion molecules, with a potentially less aggressive tumor biology and a better clinical course.^8^ Second, the more aggressive basal‐like subtype (~20%) with a better response rate to adjuvant chemotherapy.^8^ It has previously been reported that chemotherapy can induce subtype shifts, which may be of clinical relevance.^9^, ^10^

To address the tremendous chemoresistance in PDAC, we performed drug‐response analyses in vitro employing patient‐derived tumor organoids alongside analyses of induced phenotypic shifts, indicated by epithelial and epithelial–mesenchymal transition (EMT) marker expression. Pre‐therapeutic response prediction as part of an individual multimodal treatment strategy would assure the best possible outcome for patients with this aggressive disease.^11^

Over the past years, patient‐specific avatar models have become much more sophisticated and are now closely resembling the histoarchitecture, phenotypic heterogeneity, and disease stage‐specific characteristics of their primary PDAC tissue.^1^, ^12^ In general, the three‐dimensional (3D) tumor organoid technology has surfaced as a novel technique for the establishment of patient‐derived cultures to address clinically relevant questions, more closely recapitulating the disease and suitable for performing pre‐clinical drug screenings of translational value.^1^, ^2^, ^12^, ^13^, ^14^, ^15^, ^16^, ^17^, ^18^ Today, functional drug sensitivity testing (f‐DST) utilizing PDAC tumor organoids appears feasible and holds the potential of studying inter‐individual differences and personalized response predictions.^19^

With this experimental pilot study, we aimed to develop a f‐DST for PDAC in support of future clinical trials for improving current standards for neoadjuvant and adjuvant treatment strategies.

## 2 MATERIALS AND METHODS

### 2.1 Study design and patient enrolment

This correlative experimental pilot study was designed to establish the methodology of PDAC patient‐derived tumor organoid cultures at our institution. Cultures displaying sustained growth were utilized for developing a f‐DST assay based on the IndiTreat® technology of 2cureX.^20^

Tissue samples were collected between February 2020 and August 2021.

### 2.2 Participant selection

1. Enrolment of patients with histologically confirmed or suspected pancreatic cancer undergoing surgical resection of their primary tumor or metastasis.
2. Enrolment of palliative care patients with histologically confirmed pancreatic cancer undergoing treatment, including bioptic sampling of a liver metastasis.

### 2.3 Tumor organoid generation and culture

Resected tumor samples were collected in phosphate‐buffered saline (PBS) supplemented with antibiotics and processed after 2–18 h. Weights were recorded and samples were kept at 4°C until processing. Bioptic tumor samples were collected in DMEM‐based sampling media supplemented with antibiotics and bovine serum albumin (BSA). Biopsies were obtained with an 18G core needle and processed immediately upon receipt. Ultrasound‐guided or CT‐guided tumor tissue sampling was performed as part of routine diagnostic procedures, with an additional core taken for tumor organoid generation.

Tumor organoid cultures were established as initially described by Huang et al.^1^ In brief, tumor samples were cut with two Cutfix® scalpels. For resected samples, the tissue was digested with 1 mg/mL Collagenase/Dispase, and partially digested tissue fragments were transferred to a conical tube, diluted with 0.1% BSA in PBS, and centrifuged at 450*g* for 5 min. The resulting pellet was further digested with Accutase for 30 min and subsequently diluted with 0.5% BSA in Dulbecco's Modified Eagle Medium (DMEM). The digest was consecutively passed over a 100 μm and a 30 μm cell strainer. The resulting fractions were collected separately and centrifuged at 450*g* for 5 min. If needed, red blood cells present in the <30 μm fraction or in minced bioptic samples were lysed using Ammonium‐chloride‐potassium (ACK) lysing buffer according to standard protocol.

Extracted cohesive cell clusters were resuspended in 5% growth factor reduced (GFR) Matrigel® in complete Pancreas Tumor Organoid Growth Media containing Y‐27632, retinoic acid, and growth factors including FGF2 and insulin. Cell clusters sized 30–100 and <30 μm were seeded onto 24‐well plates precoated with GFR Matrigel® and cultured at 37°C and 5% CO_2_. The culture media was replaced every 3–4 days until tumor organoids reached a diameter of >200 μm and were propagated via partial enzymatic digestion (Accutase) and mechanical shearing. Subsequent tumor organoid passages and fragmented bioptic samples were cultured in GFR Matrigel® domes.

### 2.4 Subtype analysis: Gene expression analysis of tumor organoids (PurIST score)

Frozen tumor organoids were thawed, washed twice with PBS after removing the freezing medium, and total ribonucleic acid (RNA) was extracted by adding 700 μL of QiAzol Reagent and incubating it for 5 min at room temperature. Then, 140 μL of chloroform was added, and samples were mixed by vigorous vortexing for 15 s, followed by a 3‐min incubation at room temperature. Next, samples were centrifuged, and the aqueous phase was transferred to a new tube containing an equal volume of isopropanol. Samples were incubated for 1 h at −80°C, centrifuged, and RNA pellets were washed twice with ice‐cold 70% ethanol, air‐dried for 5 min at room temperature, and resuspended in 40 μL of RNase‐free water. RNA concentration was then measured using the Nanodrop, and 500 ng of RNA was used for complementary DNA (cDNA) synthesis using qScript cDNA SuperMix (Quantabio, 95048) following the manufacturer's instructions. cDNA was then diluted, and real‐time quantitative Polymerase Chain Reaction (PCR) (qPCR) was performed in duplicate for each sample using SsoAdvanced Universal SYBR Green SuperMix (Bio‐Rad, 1,725,274) on a CFX Duet Real‐Time PCR System (Bio‐Rad). All primers used are listed in Table S1.

#### 2.4.1 PurIST score

Calculation of the PurIST score for subtype analysis was adapted from the original paper by Rashid et al.^21^ Briefly, expression levels of classical and basal PDAC signature genes were determined by qPCR. Then, a specific Top Scoring Pair (TSP) vector of 0 or 1 was assigned to each sample based on a pairwise comparison of the Cq values between a basal and classical gene. Hence, if the expression level of the basal gene is higher than the classical one, a TSP vector of 1 was assigned to that sample, whereas a TSP vector of 0 was assigned when otherwise. For each pairwise comparison, a weighted TSP score was calculated by multiplying the TSP vector by the specific *β* TSP coefficient (Table S2). The final PurIST score for each sample was calculated as the sum of all weighted TSP scores from each pairwise comparison and adjusting for the model intercept (see formula). A positive PurIST score denotes a basal subtype, whereas a negative score suggests a classical one.

$$\text{PurIST score}=\mathrm{SUM}\;\left(\text{weighted TSPs}\right)+\hat{\beta }.$$

$$\hat{\beta }=-6.81.$$

### 2.5 Drug screening assays

Tumor organoids were collected, purified using Matrigel® Recovery Solution, and centrifuged at 450*g* for 5 min. Tumor organoids were resuspended in 0.5% BSA in DMEM and consecutively passed over 70 and 40 μm cell strainers. The 40–70 μm fraction was collected and centrifuged at 450 g for 5 min. The supernatant was discarded, and tumor organoids were resuspended in Pancreas Tumor Organoid Growth Media without Y‐27632. For drug screenings, ~60 tumor organoids were pipetted per well of a 384‐well flat bottom μClear® CELLSTAR® microtiter plate pre‐loaded with GFR Matrigel® containing single drugs or drug combinations. Tumor organoids were allowed to settle prior to solidifying the GFR Matrigel® at 37°C. Wells were topped up with Pancreas Tumor Organoid Growth Media and Y‐27632 to a final concentration of 5 μM and imaged using a Cytation 1 imaging multimodal reader (BioTek). Tumor organoid cultures were followed for 5 days, and endpoint analysis was performed using the CellTiter‐Glo® 3D Cell Viability Assay (Promega, G9681) according to the manufacturer's instructions. ATP as an indicator of cell viability was measured using a Synergy multimodal reader (BioTek).

### 2.6 Drug sensitivity analysis

Single concentration array plates were designed based on average IC50 values of Gemcitabine, Gemcitabine + Paclitaxel, FOLFIRINOX, Cisplatin, and Olaparib, as determined via dose‐titration assays (Methods, Supporting Information S1). These arrays were used to screen for drug sensitivities of three tumor organoid cultures not previously tested as part of dose‐titration experiments (six technical replicates per drug or drug combination tested).

For comparing drug sensitivities of tumor organoid cultures, drug screening results were plotted as % cell viability relative to the average viability of untreated negative controls (set to 100%; 12 technical replicates). For tumor organoid lines tested on dose‐titration array plates, % cell viability at compound concentrations as indicated in Table S3 was extrapolated from fitted dose–response curves.

Resulting data sets were plotted by drug (combination), ranking tumor organoid cultures according to their sensitivity to the indicated treatment, and grouped into three sensitivity categories (low, medium, and high) by calculating the mean of each data set and placing the dividers at mean ± standard deviation.

### 2.7 Clinical outcomes

Patients included in this pilot study were followed for a median time of 13 months, recording recurrence‐free survival (RFS) and overall survival (OS). Survival data and clinicopathological characteristics of patients' tumors (including G‐Status [differentiation grading] and the T‐, N‐, M‐stages) were correlated in SPSS using univariate and multivariate analyses. Results are presented as hazard ratio (HR) and 95% confidence interval (CI). Significant statements refer to *p* values of two‐tailed tests that were <.05.

A more elaborate clinical follow‐up including details on adjuvant and palliative treatment outcomes was performed for a subset of 10 patients, for which in vitro f‐DST data was obtained on matched tumor organoids.

## 3 RESULTS

### 3.1 Tumor organoid cultures can be derived and maintained from resected and bioptic samples

Of *n* = 88 patients enrolled in this pilot study, *n* = 67 presented with histologically confirmed PDAC, including tumors of classical, heterogeneous, and basal subtype (Methods, Supporting Information S1 and Figure S1). Patients were treated according to standard of care, while tissue samples were further processed for tumor organoid generation and f‐DST. Patient‐specific cultures were established, maintained, and evaluated to determine culture success rates (Table 1).

**TABLE 1:** Success rates of tumor organoid cultures. This table provides quantitative data along the process of tumor organoid generation of patients with histopathologically confirmed pancreatic cancer.

	Resected tissue (*n*)	Liver biopsy (*n*)	All (*n*)	Percentage of successful growth (*x*/*n* confirmed PDAC)
Enrolment	82	6	88	
Patients with confirmed PDAC after histopathological analysis	61	6	67	
Initial tumor organoid formation	45	4	49	73.1% (49/67)
No/insufficient viable cells or inappropriate transport buffer	15	2		
Contamination	1	0		
Tumor organoid cultures established (≥passage #2)	44	4	48	71.6% (48/67)
Tumoroid disintegration in passage #1	1	0		
Tumor organoid cultures expanded (≥passage #3)	29	4	33	49.3% (33/67)
Growth stagnation/disintegration in passage #2	15	0		
Sustained growth (≥passage #4)	20	3	23	34.3% (23/67)
Growth stagnation/disintegration in passage #3	9	3		
Tumor organoid cultures screened	9	1		

Abbreviation: PDAC, pancreatic ductal adenocarcinoma.

**FIGURE 1:**
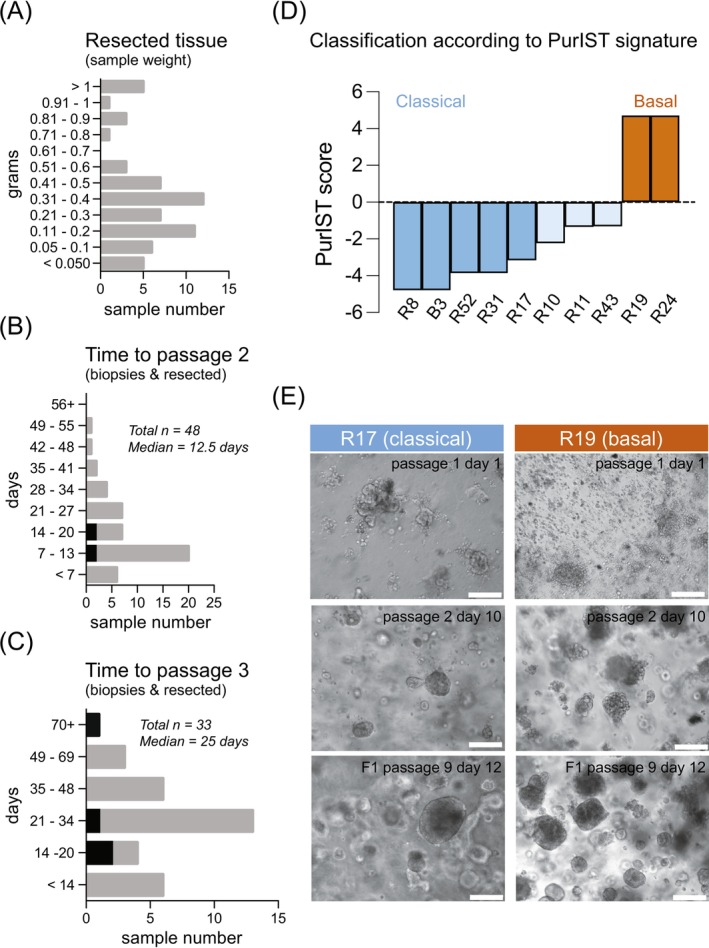
Characteristics of collected samples and derived tumor organoid cultures. (A) Sample weight of resected tissues varied from below 0.05 g to more than 1 g, with a median sample weight of 0.31 g (distribution shown). (B) Tumor organoid cultures of 48 patients could be established, reaching passage 2 with a median of 12.5 days (distribution is shown, biopsies indicated in red). (C) Tumor organoid cultures of 33 patients could further be expanded, reaching passage 3 with a median of 25 days since preparation (distribution is shown, biopsies indicated in red). (D) Classification according to PurIST signature. (E) Representative bright field images are shown of two different tumor organoid cultures of different subtypes, as determined by PurIST. Light and dark blue = classical subtype, orange = basal subtype. Scale bar = 200 μm. Brightness and contrast of bright field images were enhanced for visualization purposes. Original picture files were submitted to the journal and are available upon reasonable request.

Twenty‐six of 67 (39%) PDAC tumor specimens could not be processed on the day of sampling and were stored overnight at 4°C. The weight of samples collected from resected PDAC tumors (*n* = 61) ranged from <0.05 to >1 g, with a median weight of 0.31 g (Figure 1A). Neither overnight storage (yes/no) nor sample weight correlated with any of the recorded culture success parameters (Table S4).

Collected tissue samples of PDAC patients were analyzed regarding initial tumor organoid formation, culture establishment (≥passage 2), culture expansion (≥passage 3), and sustained growth (≥passage 4). The initial formation of tumor organoids was successful in 73.1% (49/67) of cases, with 34.3% of cultures (23/67) showing sustained growth. The culture time until passage 2 ranged between one and 54 days, with a median of 12.5 days (Figure 1B). Passage 3 was reached after an additional 4–54 days (median of 13 days) and a total of five to 71 days since preparation (median of 25 days) (Figure 1C).

### 3.2 Tumor organoids of both basal and classical molecular subtypes can be maintained and mirror their primary tumors

To better characterize patient tissue‐derived tumor organoids, the PurIST score was determined for a selection of 10 sustained and biobanked tumor organoid lines (expanded beyond passage 4), for which f‐DST and patient follow‐up were obtained. Considering the PurIST signature, 20% (2/10) of samples were classified as basal, 50% (5/10) classified as classical, and 30% (3/10) showed a classical/heterogeneous subtype, demonstrating the prevalence of tumor organoid lines with a classical subtype in this cohort (Figure 1D). Cultures of classical subtype displayed smooth and compact tumor organoids, while cultures of basal subtype were composed of more loosely associated tumor organoids, as shown in representative brightfield images of cultures R17 and R19 (Figure 1E).

Comparing the molecular subtype of analyzed tumor organoid lines to the histology of their primary tumors (Figure S1), we found that in 8/9 cases, protein expression patterns of KRT5/6 (basal marker protein) and Galectin4 (classical marker protein) mirrored the PurIST results (Table 2).

**TABLE 2:** Subtype analysis. Subtype analysis of tumor tissue and tumor organoids.

Patient ID	Tumor tissue		Tumor organoids
	KRT5/6, Galectin4 (IF)		PurIST score
R8	KRT5/6+, partial coexpression with Galectin4	Mixed (basal)	Classical
R10	Galectin4+, spotted expression of KRT5/6	Mixed (classical)	Classical
R11	Coexpression of KRT5/6 and Galectin4	Mixed	Classical
R17	Expression of KRT5/6 and Galectin4 in different areas	Mixed (classical)	Classical
R19	100% KRT5/6	Basal	Basal
R24	100% KRT5/6	Basal	Basal
R31	Galectin4+, partial coexpression with KRT5/6	Mixed (classical)	Classical
R43	Galectin4+, spotted expression of KRT5/6	Mixed (classical)	Classical
R52	100% Galectin4+, spotted expression of KRT5/6	Classical	Classical
B3	Necrotic		Classical

*Note*: The significance of colors indicates the Light blue = mixed (classical) subtype, dark blue = classical subtype, light orange = mixed (basal) subtype, orange = basal subtype, brown = basal subtype.

### 3.3 Clinicopathological data and in vitro tumor organoid growth parameters associate with survival

Of *n* = 67 PDAC patients included in the pilot study, *n* = 42 patients underwent curative tumor resection and *n* = 25 palliative patients underwent tissue analysis for diagnostic purposes (*n* = 6 via liver biopsies and *n* = 19 via surgical tissue analysis). Table S5 describes the clinicopathological data of the patient cohort.

**FIGURE 2:**
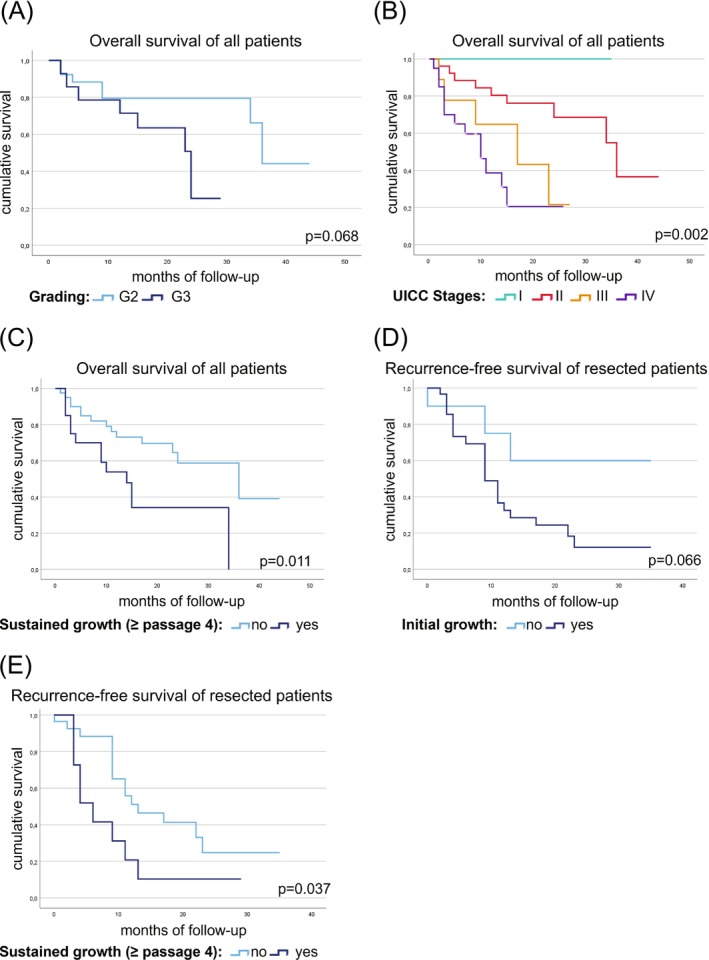
Kaplan–Meier plots. Data depict overall survival (OS) of pancreatic cancer patients (*n* = 62) in relation to (A) grading, (B) UICC stages, and (C) sustained growth of patient‐derived tumor organoid cultures. Five patients with a Clavien‐Dindo grade of 5 were excluded from OS analyses. Additional plots depict recurrence‐free survival (RFS) of pancreatic cancer patients operated with curative intent (*n* = 42) in relation to (D) initial establishment of patient‐derived tumor organoids (passage #2 reached) and (E) sustained growth of tumor organoid cultures.

We analyzed the impact of clinicopathological and tumor organoid culture success parameters on OS (Table S6). The reported OS data underlined the representativity of our cohort, with shorter OS being associated with lower differentiation grading (G3) (*p* = .068, 24 vs. 36 months; HR 3.3), higher UICC stages (*p* = .002) (or *p* < .001 when comparing UICC stages I + II versus III + IV [11 vs. 24 months; HR 14.6]) (Figure 2A,B), lymph node involvement (*p* = .046 [24 vs. 30.7 months; HR 4.0]), and distant metastasis at the time point of diagnosis (*p* < .001 [10 vs. 36 months; HR 15.2]). In this pilot study, short OS was associated with sustained tumor organoid growth (*p* = .011, 14 vs. 36 months; HR 6.5) (Figure 2C).

Our statistical analysis also indicated shorter RFS relating to higher UICC stages (I–II vs. III) (*p* = .027 [6 vs. 12 months; HR 4.9]) (Table S7). Median RFS was found to associate with initial tumor organoid growth (*p* = .066 [9 vs. 24.3 months; HR 3.4]), as well as sustained growth (*p* = .037 [6 vs. 13 months; HR 4.4]) (Figure 2D,E; Table S7).

In a multivariate analysis, sustained tumor organoid growth was not shown to be an independent prognostic factor for neither OS nor RFS (Table S8).

### 3.4 Development of an f‐DST assay based on the IndiTreat® technology

Tumor organoid cultures displaying sustained growth were used for f‐DST in a 384‐well format. An initial set of six cultures was plated and visually followed over 7 days (Methods, Supporting Information S1 and Figure S2A). Growth kinetics of these cultures varied with fold increases of tumor organoid areas ranging from 1.5 to 9.2 (average fold increase of 3.9) (Figure S2B). To avoid growth stagnation of densely seeded or fast‐growing tumor organoid cultures, we decided to shorten the duration of the assay to 5 days, for which our initial data set reported an average fold increase of 2.7.

**FIGURE 3:**
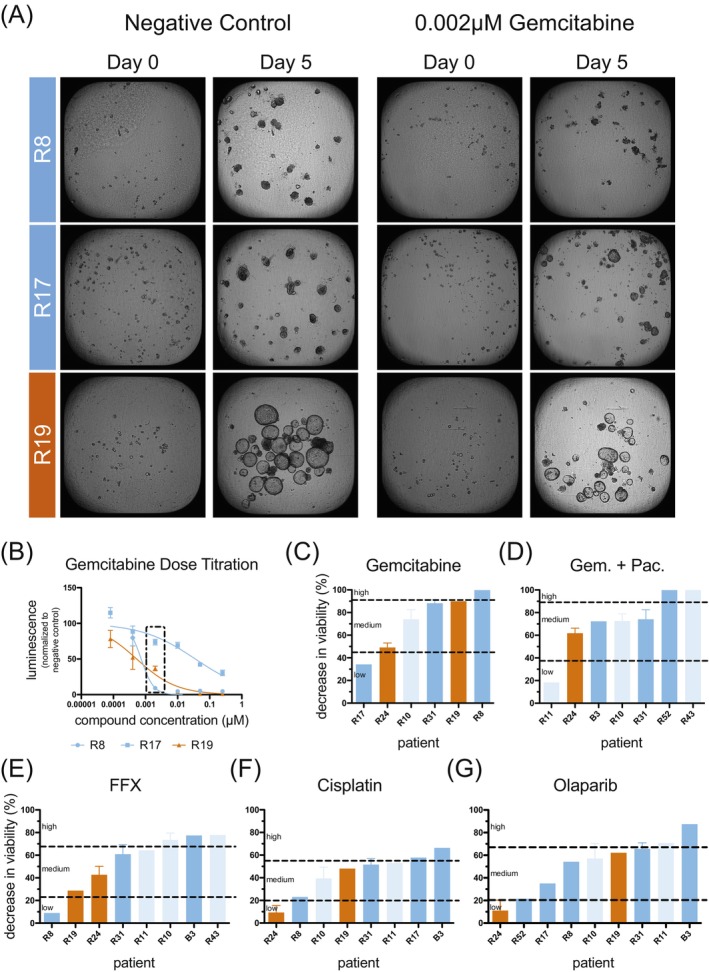
Functional drug sensitivity testing of tumor organoids. (A) Representative brightfield images of three different tumor organoid cultures, representing different subtypes, cultured to a size of 40–70 μm, seeded onto a 384‐well microarray plate pre‐loaded with Matrigel® and settled to enable microscopic analysis. Where indicated, cultures were supplemented with Gemcitabine and tumor organoid growth/growth stagnation/disintegration was followed until Day 5. (B) Endpoint analysis using the CellTiter‐Glo® 3D Cell Viability Assay was performed to record dose–response curves for Gemcitabine (*n* = 5 technical replicates/data point) of three tumor organoid cultures. (C) Using IndiTreat® Single Concentration assay plates, Gemcitabine sensitivity data were acquired for additional cultures via CellTiter‐Glo® endpoint analysis. The decrease in viability relative to the control was extrapolated from dose‐titration curves (no standard error plotted) or IndiTreat® Single Concentration screens (*n* = 6 technical replicates). Sensitivity categories were assigned by calculating the mean of the data set and placing the dividers at mean ± standard deviation to structure the cohort. Similarly, compiled functional drug sensitivity testing data for (D) Gemcitabine + Paclitaxel (Gem. + Pac.), (E) FOLFIRINOX (FFX), (F) Cisplatin and (G) Olaparib are shown. Light and dark blue = classical subtype, orange = basal subtype.

To determine IC50 values of tumor organoid cultures when exposed to clinically relevant drugs and drug combinations, we performed dose‐titration experiments on several tumor organoid cultures per compound (combination) (Methods, Supporting Information S1). Exemplarily, Figure 3A depicts tumor organoids of three patients cultured in the absence of any drug (negative control) or in the presence of 0.002 μM Gemcitabine, displaying different degrees of growth reduction/tumor organoid disintegration. Dose‐titration drug screenings were terminated on Day 5, and end point analysis of metabolically active cells was performed (luminescence readout). Screening results were obtained and plotted for Gemcitabine (Figure 3B) and other commonly used chemotherapeutics in PDAC patient care (FOLFIRINOX, Gemcitabine + Paclitaxel), as well as the less frequently used drugs (Cisplatin, Olaparib) (Figure S3). Average IC50 values derived from these curves (Table S3) formed the basis for the development of the IndiTreat® Single Concentration Array.

### 3.5 Tumor organoid cultures show marked differences in drug responses, which correlate with clinical outcomes

f‐DST employing dose‐titration assays or the IndiTreat® Single Concentration Array resulted in f‐DST data sets for tumor organoid cultures of *n* = 10 PDAC patients. Individual sensitivities to screened drugs and combinations were highly variable among cultures, ranging from <10% decrease in viability (R8 response to FOLFIRINOX, R18 response to Gemcitabine + Paclitaxel, R24 responses to Cisplatin and Olaparib) to >90% decrease in viability (R8 response to Gemcitabine, R43 response to Gemcitabine + Paclitaxel and R52 response to Gemcitabine + Paclitaxel) (Figure 3C–G). Only on a few occasions did patient‐derived tumor organoid cultures display the same sensitivity to all tested treatments (observed for cultures R31 and R19) hinting at the clinical value of the f‐DST technology for individualizing treatment plans.

In a parallel set of experiments for phenotypic epithelial and EMT marker analyses based on EpCAM and Vimentin expression, chemotherapy‐treated cultures yielding sufficient tumor organoids (*n* ≥ 15, at least 20% of baseline results shown in Table S9) were IF stained and EpCAM and Vimentin marker expression was analyzed (Methods, Supporting Information S1 and Table S9).^22^, ^23^ Tumor organoids of patient R24, while displaying a basal‐like profile at baseline, showed a diverging marker expression after exposure to any of the tested chemotherapies, with EpCAM expressed in up to 100% of treated and analyzed tumor organoids (Figure S4, Table S9).

The clinicopathological characteristics and survival data of the patient sub‐cohort for which f‐DST results were obtained are described in Table 3. RFS could be evaluated in 5 out of 10 IndiTreat®‐tested patients (due to early deaths [*n* = 4; caused by complications] and one loss of follow‐up): Patients R8 and R31 relapsed 4 months after adjuvant treatment with FOLFIRINOX or FOLFOX, respectively. For these two patients, IndiTreat® testing of derived tumor organoid cultures indicated low–medium sensitivity to the combination of 5‐FU, leucovorin, oxaliplatin, and SN‐38 (active metabolite of irinotecan). Patient R43, also treated with adjuvant FOLFIRINOX, presented with a RFS of 9 months. For this patient, f‐DST testing of patient‐specific tumor organoids indicated high sensitivity to this drug combination. Similarly, the tumor organoid culture of patient R10 displayed high sensitivity to FOLFIRINOX with the patient, however, relapsing 3 months after the initiation of adjuvant therapy. Unfortunately, data regarding therapy compliance was not available for this patient. Expanding our view from adjuvant to palliative treatment, we found that treatment of patients R8 and R24, and R31 with Gemcitabine alone or in combination with nabPaclitaxel resulted in stable disease in all three patients, with respective tumor organoid cultures displaying medium–high sensitivity to Gemcitabine‐based therapies.

**TABLE 3:** Sub‐cohort of IndiTreat®‐tested patients. The table provides detailed clinical data along the treatment journey of every patient and a comparison with acquired in vitro data. Two patients were already palliative at diagnosis (marked in light pink) and three patients died prematurely of other causes (marked in pink).

	Clinicopathological characteristics and follow‐up									Tumor organoid characteristics		
Patient ID	Age, gender, ECOG	Neoadjuvant treatment	Localization and procedure	UICC stage	G, TNM, R	Adjuvant treatment	Recurrence	Palliative treatment	Tumor‐related death	Subtype (PurIST signature)	IndiTreat® f‐DST for	In vitro drug sensitivity
R8	65 y., ♀, 0	6× FFX	Head, Whipple procedure	2	ypT2 N1 M0; R0 (CRM−)	5× FFX	Yes (after 4 mos.)	^a^	Yes (after 15 mos.)	Classical	Gem./FFX/Cisplatin/Olaparib/	High/low/medium/medium/
R10	66 y., ♂, 0	–	Head, Whipple procedure	2	G2 pT2 N1 M0; R0 (CRM−)	FFX (no data regarding therapy compliance)	Yes (after 3 mos.)	Yes, no data regarding compound	Yes (after 4 mos.)	Classical	Gem./Gem. + Pac./FFX/Cisplatin/Olaparib/	Medium/medium/high/medium/medium
R11	83 y., ♂, 1	–	Head, palliative histological analysis	4	cT4 Nx M1 (hep)	–	Palliative case at diagnosis	Not started before death	Yes (after 3 mos.)	Classical	Gem. + Pac./FFX/Cisplatin/Olaparib	Low/medium/medium/high
R17	50 y., ♂, 1	–	Head, Whipple procedure	3	G3 pT2 N2 M0; R0 (CRM−)	Not started before death	No (after 2 mos.)	–	No (death from hypoglycemia)	Classical	Gem./Cisplatin/Olaparib	Low/high/medium
R19	62 y., ♀, 1	–	Head, Whipple procedure	2	G2 pT2 N1 M0; R1	1× mFFX (stopped due to hepatic encephalopathy → palliative situation)	No (after 2 mos.)	–	No (death from liver cirrhosis, hepatic encephalopathy and upper GI bleeding)	Basal	Gem./FFX/Cisplatin/Olaparib	Medium/medium/medium/medium
R24	68 y., ♂, 0	–	Head, Whipple procedure	3	G3 pT3 N2 M0; R1	No (delayed due to complication)	Yes (after 3 mos.)	Gem./nabPac. for at least 3 mos. after recurrence → stable disease	No (still alive at 15 mos. of follow‐up)	Basal	Gem./Gem. + Pac./FFX/Cisplatin/Olaparib	Medium/medium/medium/low/low
R31	55 y., ♀, 0	^b^	Head, total duodenosplenopancreatectomy	3	G2 ypT2 N2 M0; R0 (CRM−)	9× FOLFOX	Yes (after 4 mos.)	^c^	No (still alive at 27 mos. of follow‐up)	Classical	Gem./Gem. + Pac./FFX/Cisplatin/Olaparib	Medium/medium/medium/medium/medium
R43	92 y., ♂, 0	–	Head, pylorus‐preserving pancreaticoduodenectomy	2	G2 pT2 N1 M0; R0 (CRM−)	3× mFFX	Yes (after 9 mos.)	–	Yes (after 9 mos.)	Classical	Gem. + Pac./FFX	High/high
R52	79 y., ♀, 1	–	Head, pylorus‐preserving duodenopancreatectomy	3	G3 pT2 N2 M0; R0 (CRM−)	FFX	No (after 3 mos.)	–	No (death from septic and hemorrhagic shock	Classical	Gem. + Pac./Olaparib	High/medium
B3	60 y., ♂, 1	–	Head, palliative histological analysis	4	cT2 N1 M1 (hep)	–	Palliative case at diagnosis	FFX	No follow‐up available	Mixed	Gem. + Pac./FFX/Cisplatin/Olaparib	Medium/high/high/high

Abbreviations: FFX, FOLFIRINOX, f‐DST, functional drug sensitivity testing; Gem., Gemcitabine, mFFX, modified FOLFIRINOX, Pac., Paclitaxel; y., years; ♂, male; ♀; female, ECOG, Eastern Cooperative Oncology Group, TNM, Tumour, Node, Metastasis, G, Grading, R, residual tumor, CRM, circumferential resection margin, GI, gastrointestinal.

*Note*: The bold values in IndiTreat f‐DST for und in‐vitro drug sensitivity indicates signifiance for the grudfs received by patients and respcteive in vitr drug sensivity screening results. The table provides detailed clinical data along the treatment journey of every patient and a comparison with acquired in‐vitro data. 2 patients were already palliative at diagnosis (marked in light pink) and 3 patients died prematurely of other causes (marked in pink).

^a^ Gem./nabPac. (reduced concentration) for 3 mos. → stable disease; treatment terminated due to fatigue and complications.

^b^ 6× FFX (intolerance to irinotecan → reduced concentration as of Cycle 2).

^c^ 6× Gem./nabPac. → stable disease → progress 11 mos. after recurrence and switch to FOLFOX (only two cycles received due to anaphylactic shock in response to oxaliplatin) → switch to NAPOLI (nal‐Irinotecan) → progress 15 mos. after recurrence and reintroduction of Gem. (monotherapy) 20 mos. after initial recurrence → stable disease.

## 4 DISCUSSION

This pilot study highlights the potential of f‐DST using patient‐derived PDAC tumor organoids to personalize treatment strategies. By evaluating responses to clinically relevant drugs and combinations like FOLFIRINOX, f‐DST revealed inter‐individual variability and mirrored clinical outcomes. Our approach, which utilized fixed drug concentrations, enabled sensitivity testing even under conditions of limited tumor organoid availability, potentially fitting within clinically relevant timelines.^24^, ^25^

Timely initiation of treatment is critical for PDAC patients, with adjuvant therapy typically starting within 12 weeks post‐surgery and neoadjuvant or palliative treatment beginning immediately after diagnosis. Tumor organoid cultures must therefore be tested early (within passage 3) to ensure results inform treatment decisions without unnecessary delays. While f‐DST can refine therapeutic protocols post‐initiation, initial therapy should follow established guidelines.

Consistent with recent reports,^2^, ^14^, ^18^ our study confirmed the feasibility of generating tumor organoids from freshly resected tumors and metastatic biopsies, achieving culture establishment success rates of 62%–78%, and a subsequent derivation of expanding cultures in 41% to 66% of cases. As demonstrated in this study and reported by Grossman et al.,^18^ maintaining PDAC tumor organoid cultures in higher passages proved more difficult, resulting in a sustained growth rate of 34% and 18%, respectively.

Sustained growth was associated with advanced tumor stages (UICC III/IV) and poor survival, aligning with findings that aggressive tumors are more likely to proliferate in vitro.^26^, ^27^, ^28^ However, sustained growth was not an independent prognostic factor for survival, highlighting the need for larger cohort studies to validate these findings.

Tissue storage (overnight at 4°C) did not impact culture success, suggesting that the chosen method of tissue storage was sufficient to preserve tissue viability in the short term. To our knowledge, there are no existing studies on the effect of overnight storage on PDAC tumor organoid growth. Furthermore, our data show that mere sample abundance (measured as tissue weight) does not suffice to ensure tumor organoid culture success, which is in line with previous studies suggesting successful growth from very small amounts of tissue.^14^, ^15^, ^29^ More important than tissue quantity are the cellular composition and abundance of viable tumor cells in tissue samples.^18^, ^30^, ^31^ This suggests the suitability of tumor organoids derived from limited tissue, such as endosonographically guided fine‐needle aspirations, which may also support genetic testing when other methods are insufficient.^14^, ^15^, ^29^

Tumor organoids retained the molecular subtype of their primary tumors, with PurIST scoring identifying classical and basal subtypes.^21^, ^32^ Classical subtype organoids predominated in this cohort, and molecular profiling confirmed a strong correlation between gene expression and protein markers (e.g., KR5/6, Galectin‐4). This consistency supports the potential of tumor organoids for precision oncology, particularly as basal subtypes are associated with aggressive tumor biology and differential treatment responses. The observed coexistence of both molecular subtypes and heterogeneity, which we have also observed for EpCAM and Vimentin, might explain the observed minor variations between tumor tissue and tumor organoids.^33^

Chemotherapy‐induced phenotypic shifts, including changes in epithelial and EMT markers, were observed in one basal‐like organoid culture but not in classical subtypes. While these findings are preliminary, further subtype‐specific analyses could clarify the clinical relevance of phenotypic shifts in response to therapy.^9^, ^10^, ^34^

Our pilot study shows the limitations of a small, heterogeneous cohort for f‐DST correlations with clinical outcomes. Nevertheless, initial findings suggest that pre‐ and post‐therapeutic tumor organoid testing could provide valuable insights into neoadjuvant treatment effects. Prospective clinical trials with larger patient cohorts will be essential to validate this approach and meet regulatory requirements for broader clinical adoption.

Current 3D tumor organoid models lack stromal components, which are known to influence tumor growth, metastasis, and therapy resistance.^35^ Incorporating co‐cultures or patient‐derived tumor slices that simulate the tumor microenvironment may enhance the predictive accuracy of tumor organoid‐based f‐DST. Despite these limitations, tumor organoids remain a promising platform for pre‐therapeutic drug screening, paving the way for functional precision medicine.

By combining genomic and functional testing, precision oncology has the potential to improve survival and quality of life for PDAC patients, addressing the urgent need for more effective therapies in this aggressive disease.

## AUTHOR CONTRIBUTIONS

**Christine Nitschke:** Conceptualization; data curation; formal analysis; funding acquisition; investigation; methodology; project administration; resources; software; supervision; validation; visualization; writing – original draft; writing – review and editing. **Charline Phan:** Conceptualization; data curation; formal analysis; investigation; resources; validation; writing – review and editing. **Yara Souto:** Conceptualization; investigation; methodology; resources; visualization; writing – review and editing. **Philipp Walter:** Conceptualization; data curation; formal analysis; investigation; validation; writing – review and editing. **Mara Goetz:** Conceptualization; data curation; writing – review and editing. **Gediminas Simkus:** Conceptualization; software; writing – review and editing. **Jacob Thastrup:** Conceptualization; resources; software; writing – review and editing. **Ronald Simon:** Conceptualization; investigation; resources; writing – review and editing. **Jürgen Kupper:** Conceptualization; resources; software; writing – review and editing. **Jakob Izbicki:** Conceptualization; resources; writing – review and editing. **Steven A. Johnsen:** Conceptualization; investigation; methodology; resources; supervision; writing – review and editing. **Thilo Hackert:** Conceptualization; resources; writing – review and editing. **Marianne Sinn:** Conceptualization; supervision; validation; writing – review and editing. **Harriet Wikman:** Conceptualization; formal analysis; investigation; methodology; project administration; resources; supervision; validation; visualization; writing – original draft; writing – review and editing. **Faik G. Uzunoglu:** Conceptualization; data curation; formal analysis; funding acquisition; investigation; methodology; project administration; resources; supervision; validation; visualization; writing – original draft; writing – review and editing. **Tabea M. Sturmheit:** Conceptualization; data curation; formal analysis; funding acquisition; investigation; methodology; project administration; resources; software; supervision; validation; visualization; writing – original draft; writing – review and editing.

## FUNDING INFORMATION

Mildred Scheel Cancer Career Center funded by German Cancer Aid (Christine Nitschke) provided a Clinician Scientist position covering personnel costs and providing financial support. Werner Otto and UKE/Joachim Herz Foundation (Christine Nitschke) provided financial support.

## CONFLICT OF INTEREST STATEMENT

Employment or Leadership: Chief Technology Officer of 2cureX (Jacob Thastrup). Stock Ownership: Ownership of 2cureX stocks (Jacob Thastrup, Jürgen Kupper). All other authors declare no conflicts of interest.

## ETHICS STATEMENT

The local ethical committee approved the study, and written informed consent was obtained from all patients (PV3548, September 5, 2019). The study has been registered in the German Clinical Trials Register (DRKS00023315).

## Supporting information


**Data S1.** Supporting Information.

## Data Availability

The data that support the findings of this study are available from the corresponding author upon reasonable request.
