# Supplementary material for: Patient‐derived tumor organoids highlight the potential of precision medicine in managing pancreatic ductal adenocarcinoma
Source: Int J Cancer. 2025 Apr 28;157(4):760–72. doi: 10.1002/ijc.35443 (PMC12178096; doi:10.1002/ijc.35443)
Supplement: Supplementary file 1 — Data S1. Supporting Information. [file IJC-157-760-s001.pdf]

Patient-derived tumor organoids highlight the potential of precision medicine in managing pancreatic ductal adenocarcinoma

Authors: Christine Nitschke, Charline Phan, Yara Souto, Philipp Walter, Mara Goetz, Gediminas Simkus, Jacob Thastrup, Ronald Simon, Jürgen Kupper, Jakob Izbicki, Steven A. Johnsen, Thilo Hackert, Marianne Sinn, Harriet Wikman, Faik G. Uzunoglu and Tabea M. Sturmheit

Table of contents:

Supplementary Materials & Methods

- Immunofluorescene stainings of tumor tissue samples
- Tumor organoid growth analysis
- Array design
- Immunofluorescence (IF) staining and phenotypic analysis of tumor organoids

Supplementary tables

- Supplementary Table 1: List of primers.
- Supplementary Table 2: Pairwise comparison for PurIST score.
- Supplementary Table 3: IC50 values of individual screens and adjusted r2 values of fitted curves.
- Supplementary Table 4: Correlation of tumoroid growth and tissue sampling.
- Supplementary Table 5: Patient Cohort.
- Supplementary Table 6: Univariate Overall Survival analysis.
- Supplementary Table 7: Univariate Recurrence-free Survival analysis.
- Supplementary Table 8: Multivariate analysis.
- Supplementary Table 9: Epithelial & EMT marker analysis.

Supplementary figures

- Supplementary Figure 1: Immunohistochemical analysis of tumor tissue
- Supplementary Figure 2: Growth analysis of tumor organoids
- Supplementary Figure 3: Chemosensitivity testing of tumor organoids
- Supplementary Figure 4: Chemotherapy-induced shift of basal tumor organoids (patient R24)
- Supplementary Figure 5: EpCAM Wishbone plot

Supplementary Methods:

Immunofluorescene stainings of tumor tissue samples

FFPE embedded pancreatic tumor tissue were analyzed for αSMA, LGALS4 (Galectin4), and KRT5/6 expression using a multiplex immunofluorescence staining using the Opal manual detection kit (Akoya Biosciences, NEL861001KT) following the manufacturer's protocol. Briefly, 2,5μm tissue sections were deparaffinized, rehydrated, subjected to heat-induced epitope retrieval and incubated with primary antibodies αSMA (Abcam, ab5694, dilution 1:200), anti-KRT5/6 (Abcam, ab17133, dilution 1:25) and anti-LGALS4 (Sigma, HPA031184, dilution 1:50). The following tyramide dyes from the Opal Detection kit were used for antibody visualization: Opal Polaris 480, Opal 620, and Opal 690. Slides were then mounted with ProLong Diamond Antifade Mountant (ThermoFisher Scientific, P36961) and imaged with a PhenoImager Fusion system (Akoya Biosciences). αSMA was used to visualize the stroma. Accordingly, tumor tissue expressing 100% KRT5/6 was categorized as basal, while Galectin4 expression was categorized as classical; a coexpression was considered heterogenous (mixed) subtype. Brightness and contrast of images were enhanced for visualization purposes. Original picture files were submitted to the journal and are available upon reasonable request.

Tumor organoid growth analysis

Growth kinetics were evaluated of tumor organoid cultures displaying sustained growth (≥ passage 4). Tumor organoids were grown to an approximate size of 70μm-100μm in diameter, collected into a conical tube and centrifuged at 450g for 5 minutes. The supernatant was discarded and residual GFR Matrigel® was dissolved using Matrigel® Recovery Solution. Tumor organoids were resuspended in 0.5% BSA in DMEM and consecutively passed over 100μm and 70μm cell strainers. The 70 – 100μm fraction was collected and centrifuged at 450g for 5 minutes. The supernatant was discarded and tumor organoids were resuspended in Pancreas Tumor Organoid Growth Media without Y-27632. To perform the growth analysis, ~25 tumor organoids were pipetted per well of a 384-well flat bottom μClear® CELLSTAR® microtiter plate pre-loaded with GFR Matrigel®. Tumor organoids were allowed to settle prior to moving the microtiter plate to a 37°C incubator for 30 minutes to solidify the GFR Matrigel®. Wells were

topped up with Pancreas Tumor Organoid Growth Media and Y-27632 to a final concentration of 5µM. The microtiter plate was centrifuged at 200g for 3 minutes and afterwards moved to a Cytation 1 imaging multimode reader (BioTek) for brightfield (BF) image acquisition. Tumor organoid growth was followed for a total of 7 days with additional BF images acquired on days 3, 5, 6 and 7. Additionally, an endpoint analysis was performed using the CyQUANT™ Cell Proliferation Assay (ThermoFisher). On day 6, CyQUANT™ staining solution prepared according to the manufacturer's instructions were added per well and incubated for 20-26 hours prior to day 7 image acquisition of the fluorescence signal (GFP/FITC channel).

Brightfield images of all time points and day 7 fluorescence images were analyzed using the proprietary IndiNet® image analysis algorithm, developed based on a convolutional neural network (CNN) approach. The CNN model used for image segmentation was trained on a high number of labelled BF images. The deep neural network learned to separate pixels associated with living tumor organoids from background, which was used to semi-automatically quantify viable tumor organoid areas per well over the time course of 7 days.

Array design

For dose-titration assays, 384-well flat bottom µClear® CELLSTAR® microtiter plates were preloaded with individual drugs immobilized in GFR Matrigel® in five (Cisplatin and Olaparib) to six (Gemcitabine and Paclitaxel) different concentrations. Every compound concentration was plotted with 5 technical replicates. Additionally, 10 wells were prepared with only GFR Matrigel® for negative controls. Dose-titration drug screening assays were performed on 3 to 6 tumor organoid cultures.

Tumor organoid viability after drug treatment was measured per well. The luminescence intensity of individual drug screening wells was normalized to the average luminescence intensity of all untreated control wells and % luminescence of negative control was plotted. Curves were fitted to plotted data sets using least squares regression and the goodness-of-fit was evaluated by looking at adjusted r2 values. Fitted curves were used to calculated IC50 values for tested drugs and tumor organoid lines (Suppl. Table 3). Single concentration drug screening arrays were subsequently designed based on average IC50 values of tumor organoid lines previously analysed.

To determine a fixed concentration of the Gemcitabine + Paclitaxel combination treatment, both compounds were initially titrated separately, mixed at average IC50 compound concentrations and the resulting cocktail was again titrated and tested on 5 different tumor organoid lines to determine the average IC50 value of the chemotherapy combination. Individual drug components of FOLFIRINOX (5-FU, SN38, Oxaliplatin and Leucovorin) were mixed at average IC50 concentrations previously determined on colorectal cancer tumor organoid lines (ratio of 1:0.6:0.008:1). The resulting mix was titrated on five PDAC tumor organoid lines and the average IC50 value of the combination therapy was determined.

Immunofluorescence (IF) staining and phenotypic analysis of tumor organoids

Tumor organoids were taken up in GFR Matrigel® and seeded onto 8-well chamber slides. After seven days of cultivation, tumor organoid cultures were fixed with 10% formalin for 30 minutes, washed with PBS, permeabilized with 0.2% Triton X-100 and immunostained with anti-EpCAM (Abcam, ab213500, dilution 1:100) and anti-Vimentin (Abcam, ab24525, dilution 1:200). Primary antibodies were detected with fluorescently labelled secondary antibodies (Goat Anti-Rabbit 555nm (AlexaFluor (red), dilution 1:200) + goat anti-chicken 488nm (AlexaFluor (green), dilution 1:200)). Nuclei were counterstained with Hoechst prior to applying mounting media and cover slips. Cultures with ≥ 20 IF-stained and visualizable tumor organoids were included in a phenotypic analysis based on the expression of the epithelial marker EpCAM and the EMT marker Vimentin.

As demonstrated by the EpCAM wishbone plot (Suppl. Figure 5), in pancreatic cancer, EpCAM marker expression is found in tumor cells of both subtypes, but more strongly associates with a classical differentiation path. Accordingly, cultures solely expressing EpCAM in ≥100% of tumor organoids were categorized as classical, while cultures void of EpCAM expression in tumor organoids were categorized as basal. Most cultures displayed a heterogenous EpCAM expression (between 0 and 100%) (Suppl. Table 9). Vimentin expression – considered a marker of mesenchymal differentiation – was evaluated as an indicator for EMT.

To investigate the effect of chemotherapy-induced phenotypic marker shifts of tumor organoids, lines cultured on 8-well chamber slides were exposed to either Cisplatin,

Gemcitabine + Paclitaxel or FOLFIRINOX at concentrations also used for single concentration drug sensitivity screens. Cultures were exposed to chemotherapy for a total of five days, prior to fixation, IF staining and scoring. Cultures with ≥ 15 IF-stained and visualizable tumor organoids and at least 20% of baseline counts were included in the analysis post chemotherapy.

Supplementary Tables:

Supplementary Table 1: List of primers.

Target	Forward sequence	Reverse sequence
ANXA10	CATGTATGGCCGGGACCTG	GTGGTGGTGGGTACATGAGG
BCAR3	CTGCCGATTGGCTGCAAG	CTCGCTTCCCGTCCTGAAC
C16orf74	CGTCCTGAACGACAAGCAC	GTCTCATCCAGCCAGACTGT
CLDN18	TGGAGTGTCTGTGTTTGCCA	CGCACCAAATGTGTACCTGG
CLRN3	GCTGAGCTCTGGGTTTACCT	CAAAAACGAAGGATGCACCGA
DDC	AGTCGGTCCTATCTGCAACAA	ACTCCATTCAGAAGGTGCCG
GATA6	ATCAAAGACTTGCTCTGGTAATAGC	CTCGGGATTGGTGCTCTCTC
GPR87	TCCTGACACGCATCTTTGCT	CCGTGCAGCTCGTTATTTGG
ITGA3	CCTACAACTGGAAAGGAAACAGC	CTACCTGCATCGTGTACCCA
KRT5	AGGAATGCAGACTCAGTGGAGA	CCAGAGGAAACACTGCTTGTGA
KRT6A	AGGACCTGGTGGAGGACTTC	GGCAGCATCCACATCCTTCT

LGALS4	GGAACAGCCTTCTGAATGGCTC	CCATTGGCGTAAACCTTGAAGCG
PTGES	CTGCCTCAGGGCCCAC	GTGCATCCAGGCGACAAAAG
REG4	TGCAGATCAAGGAGAACCCAG	AGCCATCTTCCTCCTACCCT
S100A2	GCAGCCTGGATGAGAACAGT	GCAGCCCTGGAAGAAGTCAT
SLC40A1	CGATAGCAGCCGCAGAAGAG	TAGTCGGCCAAGGATCCACA

Supplementary Table 2: Pairwise comparison for PurIST score.

Pairwise comparison	β TSP coefficient
GPR87 vs. *REG4*	1.99
KRT6A vs. *ANXA10*	2.03
BCAR3 vs. *GATA6*	1.62
PTGES vs. *CLDN18*	0.92
ITGA3 vs. *LGALS4*	1.06
C16orf74 vs. *DDC*	0.93
S100A2 vs. *SLC40A1*	2.51
KRT5 vs. *CLRN3*	0.46

Supplementary Table 3: <u>IC50 values of individual screens and adjusted r2 values of fitted curves.</u> Average IC50 values were calculated and used to determine single compound concentrations for the IndiTreat® Single Concentration Assay (marked in green).

**Patient ID**	**Gemcitabine**		**Paclitaxel**		**Gem. + Pac.**		**FOLFIRINOX**		**Cisplatin**		**Olaparib**	
	IC50 (µM)	*r2 adjusted*	*IC50 (µM)*	*r2 adjusted*	*IC50 (%)*	*r2 adjusted*	*IC50 (%)*	*r2 adjusted*	*IC50 (µM)*	*r2 adjusted*	*IC50 (µM)*	*r2 adjusted*
**R8**	0.000719	0.9572	0.000436	0.8301			198.4	0.5776	6.152	0.8351	12.24	0.8005
**R11**					152.2	0.5795	65.86	0.752	2.622	0.755	2.9	0.8478
**R17**	0.03558	0.7804	0.2322	0.4199					1.182	0.4106	21.24	0.5996
**R19**	0.000369	0.8016	0.05113	0.7165	199.2	0.881	189.4	0.6762	3.173	0.9371	4.673	0.8916
**B3**					47.76	0.8992	33.07	0.5925	0.3386	0.6886	2.271	0.7845
**R43**					9.267	0.7212	33.49	0.6334				
**R52**					11.62	0.7453			26.55	0.2533	37.09	0.4967
	**0.01**		**0.09**		**85**		**110**		**3**		**14**	

Supplementary Table 4: Correlation of tumoroid growth and tissue sampling.

Tissue sample		Initial growth (n (%))			Sustained growth (at least until #3) (n (%))			Sustained growth (at least until #4) (n (%))		
		No	Yes	P-value	No	Yes	P-value	No	Yes	P-value
Weight[1]	<100mg	3 (18.8)	5 (11.1)	0.825	6 (18.8)	2 (6.9)	0.148	6 (14.6)	2 (10.0)	0.558
	100-500mg	10 (62.5)	30 (66.7)		22 (68.8)	18 (62.1)		28 (68.3)	12 (60.0)	
	>500mg	3 (18.8)	10 (22.2)		4 (10.5)	9 (31.0)		7 (17.1)	6 (30.0)	
Overnight storage	No	11 (61.1)	30 (61.2)	1.000	21 (61.8)	20 (60.6)	1.000	26 (59.1)	15 (65.2)	0.793
	Yes	7 (38.9)	19 (38.8)		13 (38.2)	13 (20.4)		18 (30.9)	8 (34.8)	

[1] Weight available for n=61 out of n=67 patients (for n=6 patients missing)

Supplementary Table 5: Patient Cohort. Baseline characteristics of the patient collective with histopathologically confirmed pancreatic cancer and subdivision into the cohorts of curatively resected and palliative patients.

**Number of PDAC patients (n)**		**all patients (n=67)**	**curatively resected (n=42)**	**palliative (n=25)**
**Median age at surgery (years)**		66	67.5	62
**Gender**	male	31 (46%)	18 (43%)	13 (52%)
	female	36 (54%)	24 (57%)	12 (48%)
**Surgical approach**	NA (liver biopsy at palliative stage)	6 (9%)	/	6 (24%)
	resectable	42 (63%)	42 (100%)	/
	palliative stage at surgery	19 (28%)	/	19 (76%)
**Histological subtype**	pancreatic ductal adenocarcinoma	64 (95.5%)	40 (95%)	24 (96%)
	adenosquamous carcinoma	2 (3%)	2 (5%)	/
	colloid carcinoma	1 (1.5%)	/	1 (4%)
**Tumor localization**	pancreatic head	46 (69%)	34 (81%)	12 (48%)
	pancreatic body	11 (16%)	5 (12%)	6 (24%)
	pancreatic tail	10 (15%)	3 (7%)	7 (28%)
**G stage**	2	29 (43%)	25 (59.5%)	4 (16%)
	3	16 (24%)	14 (33.5%)	2 (8%)
	not available	22 (33%)	3 (7%)	19 (76%)
**UICC stage**	I	5 (7.5%)	5 (12%)	/
	II	29 (43.3%)	29 (69%)	/
	III	10 (14.9%)	8 (19%)	2 (8%)
	IV	23 (34%)	/	23 (92%)
**T stage**	T1	5 (7.5%)	5 (12%)	/
	T2	18 (27%)	15 (36%)	2 (12%)
	T3	25 (37%)	22 (52%)	2 (12%)
	T4	19 (28.5%)	/	19 (76%)
**N stage**	N0	16 (24%)	12 (29%)	4 (16%)

	N1	29 (43%)	22 (52%)	7 (28%)
	N2	11 (16.5%)	8 (19%)	3 (12%)
	not available	11 (16.5%)	/	11 (44%)
**M stage**	M0	44 (66%)	42 (100%)	2 (8%)
	M1	23 (34%)	/	23 (92%)
**Tissue source**	primary tumor	45 (67%)	42 (100%)	3 (12%)
	peritoneal metastasis	7 (10.5%)	/	7 (28%)
	hepatic metastasis	11 (16.5%)	/	11 (44%)
	pulmonary metastasis	4 (6%)	/	4 (16%)

Supplementary Table 6: Univariate Overall Survival analysis.

Univariate OS analysis (* if median not reached, mean was used)

OS analysis (univariate)		N=62 patients (n=5 CD5 excluded from total n=67 patients)	Median RFS, months (95% CI)	HR	P value
**Age**	< 68 years	37	23 (14.4 -31.6)	0.6	0.443
	≥ 68 years	25	34 (19.4 – 48.6)	Reference	
**Gender**	Male	27	36 (7.3 – 64.7)	Reference	0.664
	Female	35	24 (13.2 – 34.8)	0.2	
**ECOG**	0	28	23 (12.3 – 33.7)	0.01	0.993
	1	28	34 (0 – 68.7)	Reference	
	2	6	23.1 (9.9-36.3)*	-	
**UICC stage**	I / II	30	36 (32.4 – 39.6)	Reference	**<0.001**
	III/ IV	32	11 (5.8 – 16.2)	14.6	
**Tumor location in pancreas**	Caput	43	34 (10.4 – 57.6)	Reference	0.701
	Corpus	10	18.5 (4.1 – 10.5)*	-	
	Cauda	9	14 (3.9 -24.1)	0.7	
**G Status**[1]	2	26	36 (32.4 – 39.6)	Reference	***0.068***
	3	14	24 (14.9 – 33.1)	3.3	
**T-Stage**	T1 / T2	22	34 (1.0 – 67.0)	Reference	0.723
	T3 / T4	40	23 (12.1 – 33.9)	0.1	
**N-Stage**[2]	N0	14	30.7 (25.4 – 36.1)*	Reference	**0.046**
	N1 / N2	37	24 (8.2 – 40.0)	4.0	
**M-Stage**[3]	M0	41	36 (22.2 – 49.8)	Reference	**<0.001**
	M1	21	10 (5.0 – 15.0)	15.2	
**Systemic therapy**	No	3	3	7.1	**0.008**
	Yes	59	34 (16.5 – 51.5)	Reference	
**Ca19-9**	<500 U/ml	33	27.4 (21.5 – 33.2)*	Reference	***0.071***
	>=500 U/ml	25	14 (0 – 31.5)	3.3	

**Clavien Dindo (CD)**	CD 0-2	39	34 (22.8 – 45.2)	Reference	0.367
	CD 3-4	17	20.8 (13.9 – 27.7)*	0.8	
**Initial growth**	No	17	24.6 (18.0 – 31.2)*	Reference	0.450
	Yes	45	23 (6.6 – 39.4)	0.6	
**Sustained growth (at least until #3)**	No	32	36 (18.8 – 53.2)	Reference	***0.061***
	Yes	30	15 (8.9 – 21.1)	3.5	
**Sustained growth (at least until #4)**	No	41	36 (15.3 – 56.7)	Reference	**0.011**
	Yes	21	14 (8.6 – 19.4)	6.5	

[1] G status available for n=40 out of the analyzed n=62 patients (in n=22 patients missing (mostly palliative patients with only histological prove of adenocarcinoma))

[2] N status available for n=51 out of the analyzed n=62 patients (in n=11 palliative patients missing)

[3] Ca 19-9 value available for n=57 out of the analyzed n=62 patients (in n=4 palliative patients missing)

Supplementary Table 7: Univariate Recurrence-free Survival analysis.

Univariate RFS analysis (* if median not reached, mean was used)

RFS analysis (univariate)		N=42 resected patients	Median RFS, months (95% CI)	HR	P value
**Age**	< median age 68 years	21	9 (6.5 – 11.5)	1.8	0.176
	≥ median age 68 years	21	17 (6.4 – 27.6)	Reference	
**Gender**	Male	18	11 (6.4 – 15.6)	0.2	0.678
	Female	24	11 (6.9 – 15.1)	Reference	
**ECOG**	0	19	9 (7.0 – 11.0)	2.8	0.252
	1	17	17 (8.2 – 25.8)	-	
	2	6	24.7 (8.1 – 41.2)*	Reference	
**UICC stage**	I / II	34	12 (8.1 – 15.9)	Reference	**<u>0.027</u>**
	III	8	6 (1.8 -10.2)	4.9	
**Tumor location in pancreas**	Caput	34	11 (6.2 -15.8)	-	0.128
	Corpus	5	13 (9.8 -16.2)	Reference	
	Cauda	3	6.7 (2.1 – 11.2)*	4.1	
**G Status**[1]	2	25	11 (7.7 – 14.3)	0.2	0.634
	3	14	13 (9.3 – 16.7)	Reference	
**T-Stage**	T1	5	11 (1.9 – 20.1)	Reference	0.861
	T2	15	9 (3.0 – 14.8)	0.3	
	T3	22	11 (2.1 -15.1)	-	
**N-Stage**	N0	12	13 (7.1 – 18.9)	Reference	0.213
	N1	22	11 (8.4 – 13.6)	-	
	N2	8	6 (1.8 – 10.2)	3.1	
**R status**	R0 (CRM-)	22	9 (4.0 – 14.0)	0.3	0.588
	R0(CRM+)/R1	20	12 (8.4 – 15.6)	Reference	
**Neoadjuvant Therapy**	No	36	11 (7.4 – 14.6)	Reference	0.171
	Yes	6	4 (0 – 11.2)	1.9	
**Adjuvant Therapy**	No	5	2.8 (2.3 – 3.2)*	1.7	0.193

	Yes	37	11 (7.0 – 15.0)	Reference	
**Ca19-9**	<500 U/ml	27	9 (4.8 – 13.2)	0.001	0.969
	>=500 U/ml	12	13 (9.3 – 16.7)	Reference	
**Initial growth**	No	10	24.3 (14.7 – 33.9)*	Reference	***0.066***
	Yes	32	9 (6.0 – 12.0)	3.4	
**Sustained growth (at least until #3)**	No	23	11 (7.1 – 14.9)	Reference	0.372
	Yes	19	9 (2.8 – 15.2)	0.8	
**Sustained growth (at least until #4)**	No	28	13 (4.3 – 21.7)	Reference	**<u>0.037</u>**
	Yes	14	6 (3.1 – 8.9)	4.4	

[1] G status available for n=39 resected patients (in n=3 patients missing)

Supplementary Table 8: Multivariate analysis.

Overall survival

**Multivariate OS analysis**		**OS all patients**	
		**HR (95% CI)**	**P value**
**UICC stage**	I / II	Reference	**<0.001**
	III / IV	4.8 (1.9 – 11.8)	
**Sustained growth (at least until #4)**	No	Reference	0.174
	Yes	1.8 (0.8 – 4.0)	
**Systemic therapy**	No	0.2 (0.04 – 0.9)	**0.041**
	Yes	Reference	

Recurrence-free survival

**Multivariate OS analysis**		**OS all patients**	
		**HR (95% CI)**	**P value**
**UICC stage**	I / II	Reference	***0.074***
	III	2.7 (0.9 – 7.9)	
**Sustained growth (at least until #4)**	No	Reference	***0.081***
	Yes	2.2 (0.9 – 5.1)	

Supplementary Table 9: Epithelial & EMT marker analysis. IF staining results of established tumor organoid lines prior to (baseline) and following drug exposure in-vitro. Dark blue = cultures expressing EpCAM in 100% of tumor organoids (classical phenotyp), light blue = cultures expressing EpCAM in ≥ 70% of tumor organoids (mixed/classical phenotyp), orange = cultures expressing EpCAM in ≤ 30% of tumor organoids (mixed/basal phenotyp). Cis. = Cisplatin, Gem. + Pac. = Gemcitabine + Paclitaxel

**Patient ID**	**% EpCAM**					**% Vimentin**			
	baseline	Cis.	Gem. + Pac.	FFX		baseline	Cis.	Gem. + Pac.	FFX
**R8**	89	99	100			84	99	100	
**R10**	98					80			
**R11**	79					46			
**R17**	85	81	100			75	100	100	
**R19**	98	100	99	100		100	100	100	100
**R24**	19	100	96	100		37	4	57	94
**R25**	8					55			
**R30**	95	100	100	100		71	98	100	100
**R34**	82					45			
**R43**	100		100	100		80		100	100
**R52**	100	100	100	100		100	100	100	100
**B3**	96					98			

Supplementary Figure 1: Immunohistochemical analysis of tumor tissue

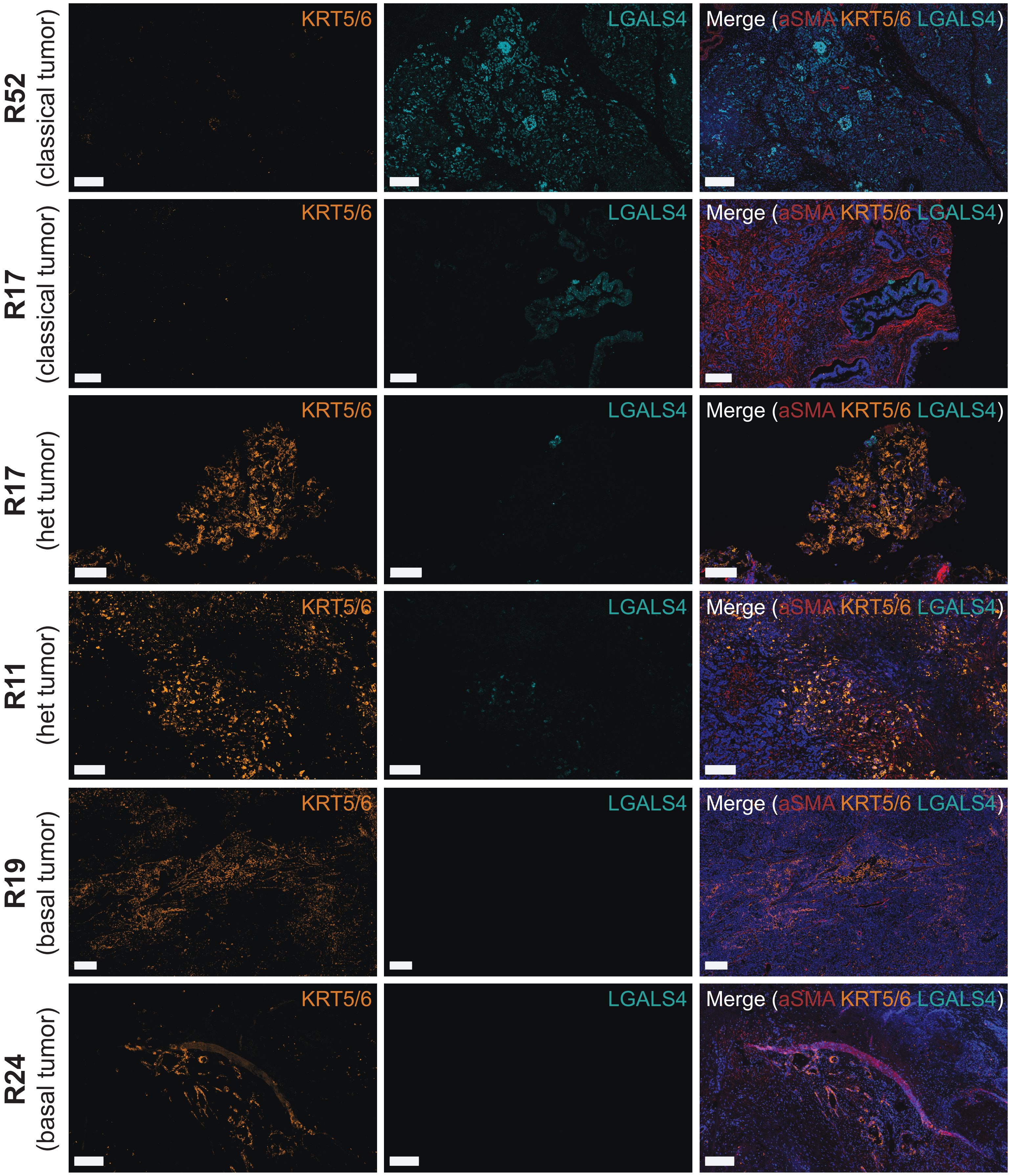

KRT5/6 (orange) displaying basal subtypes and LGALS4 (Galectin4) (turquoise) displaying classical subtypes in tumor tissue of six PDAC patients. scale bar = 200µm

Supplementary Figure 2:
Growth analysis of tumor organoids

(A)

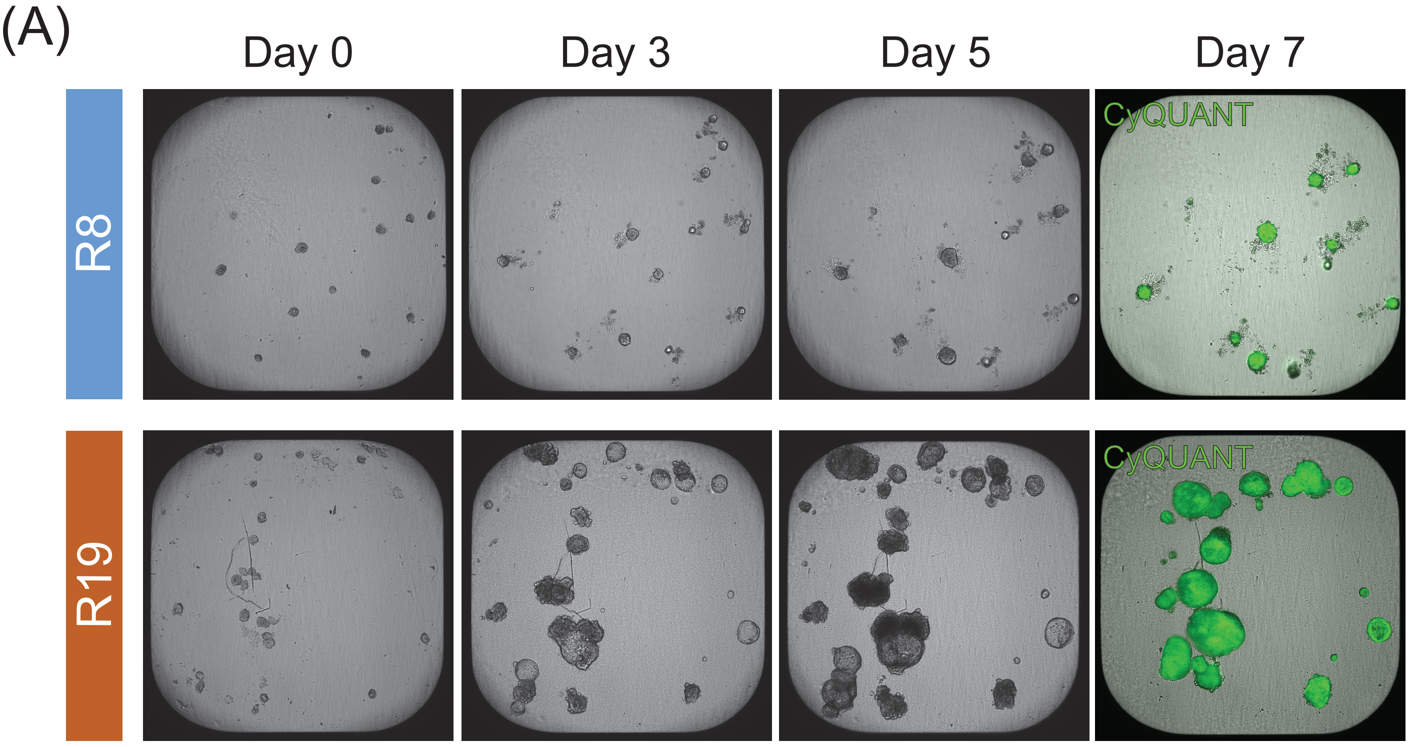

(B)

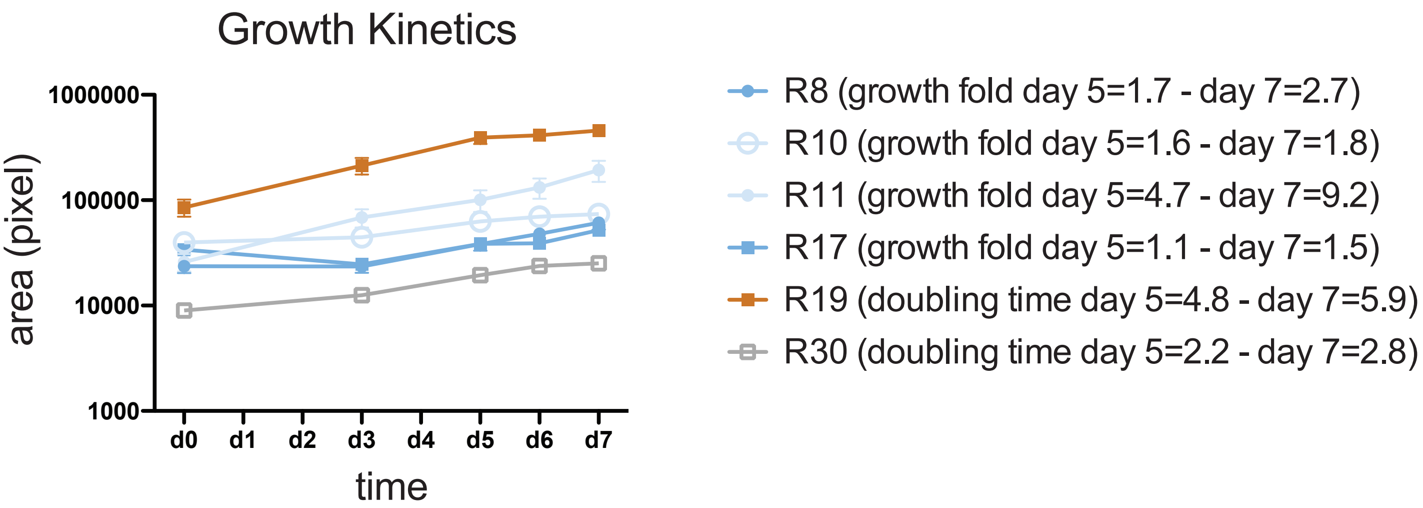

(A) Representative bright field images of 2 different tumor organoid cultures, representing different molecular subtypes according to PurIST score, cultured to a size of 70-100µm and seeded onto 384-well microarray plates pre-loaded with Matrigel® and settled to enable microscopic analysis. Tumor organoid growth was followed over 7 days and visualized with a Cytation 1 imaging reader. Additional endpoint analysis was performed using the CyQuant® Direct Cell Proliferation Assay. (B) Tumor organoid areas as shown in A were analysed using 2cureX's proprietary IndiNet® software (data points of 6 patient-derived cultures are plotted, displaying different growth kinetics (n=6-12 technical replicates / data point). Light and dark blue = classical subtype, orange = basal subtype, grey = subtype not determined

Supplementary Figure 3:
Chemosensitivity testing of tumor organoids

(A)

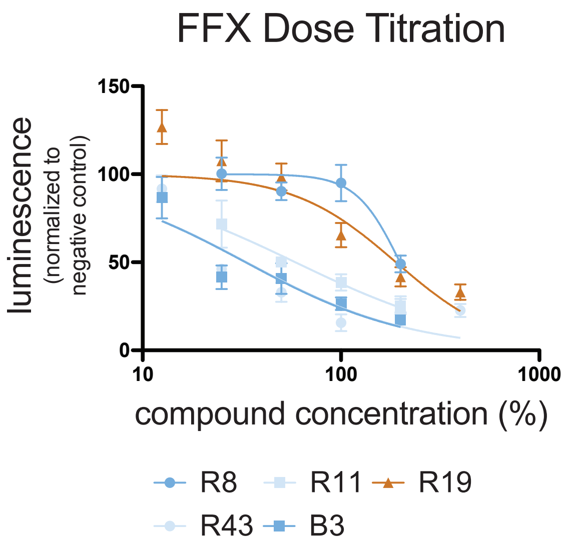

(B)

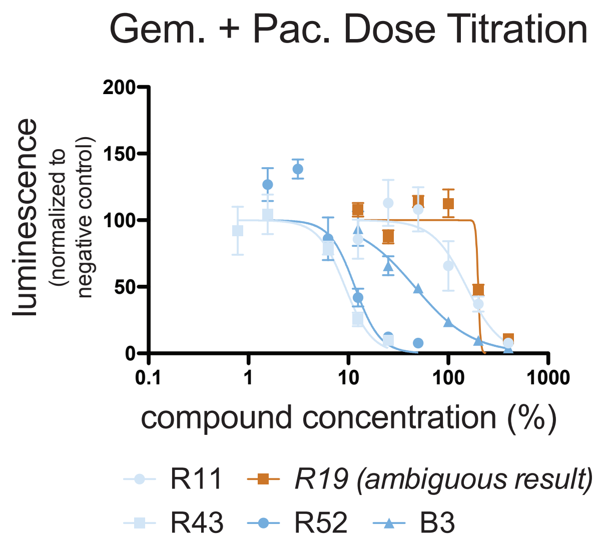

(C)

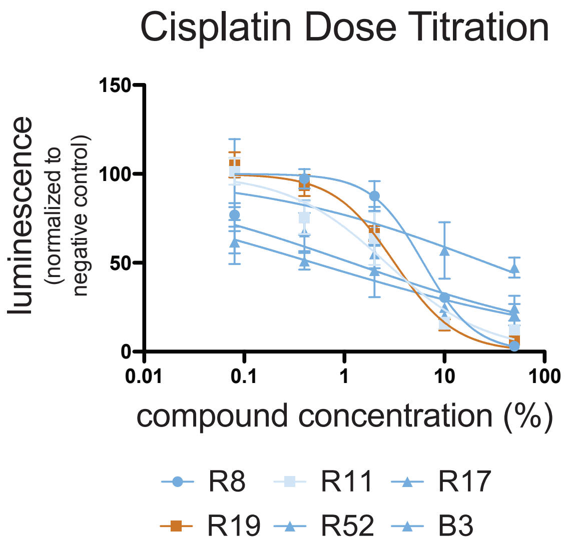

(D)

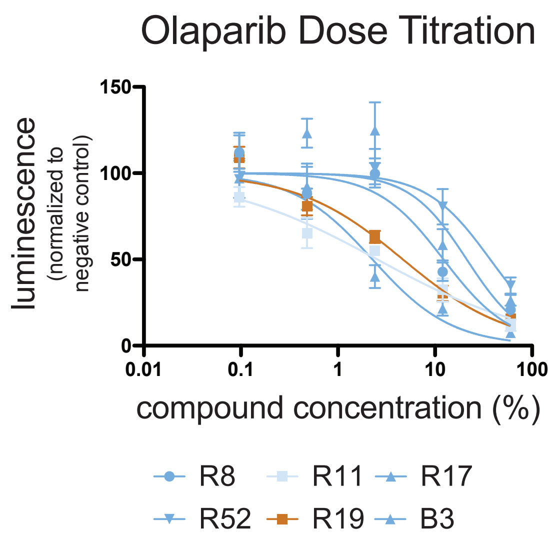

Endpoint analysis using the CellTiter-Glo® 3D Cell Viability Assay was performed to record dose-response curves for (A) FOLFIRINOX (FFX), (B) Gemcitabine + Paclitaxel (Gem. + Pac.), (C) Cisplatin and (D) Olaparib of 5 – 6 established tumor organoid cultures of different molecular subtypes (n = 5 technical replicates / data point). Light and dark blue = classical subtype, orange = basal subtype

Supplementary Figure 4:
Chemotherapy-induced shift of basal tumor organoids (patient R24)

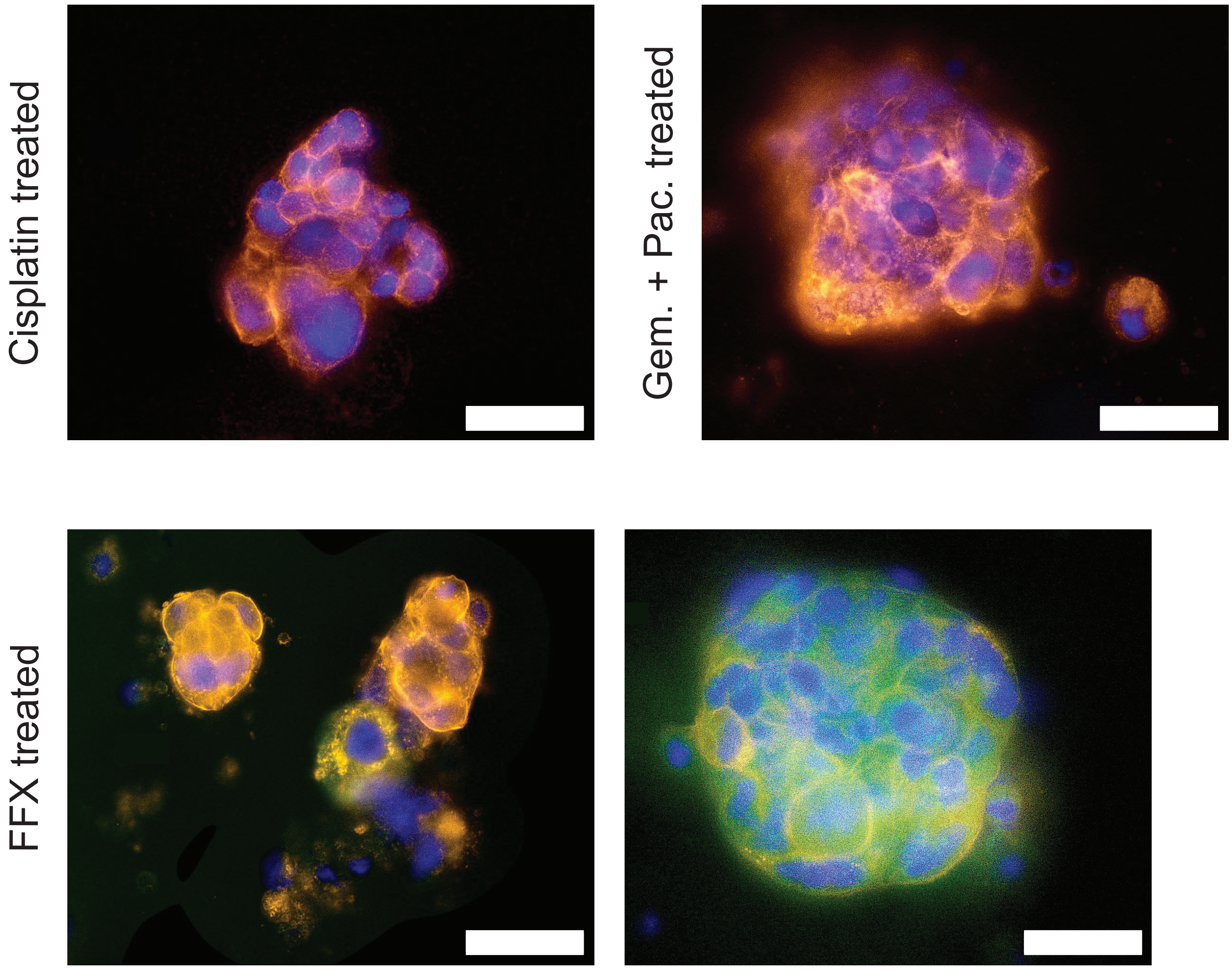

Representative immunofluorescence images of R24 tumor organoid culture (basal subtype according to PurIST score) undergoing a chemotherapy-induced shift of EpCAM and Vimentin marker expression after treatment with Cisplatin, Gemcitabine + Paclitaxel (Gem. + Pac.) or FOLFIRINOX (FFX) (see also supplementary table 9). scale bar = 100µm

Supplementary Figure 5: EpCAM Wishbone plot

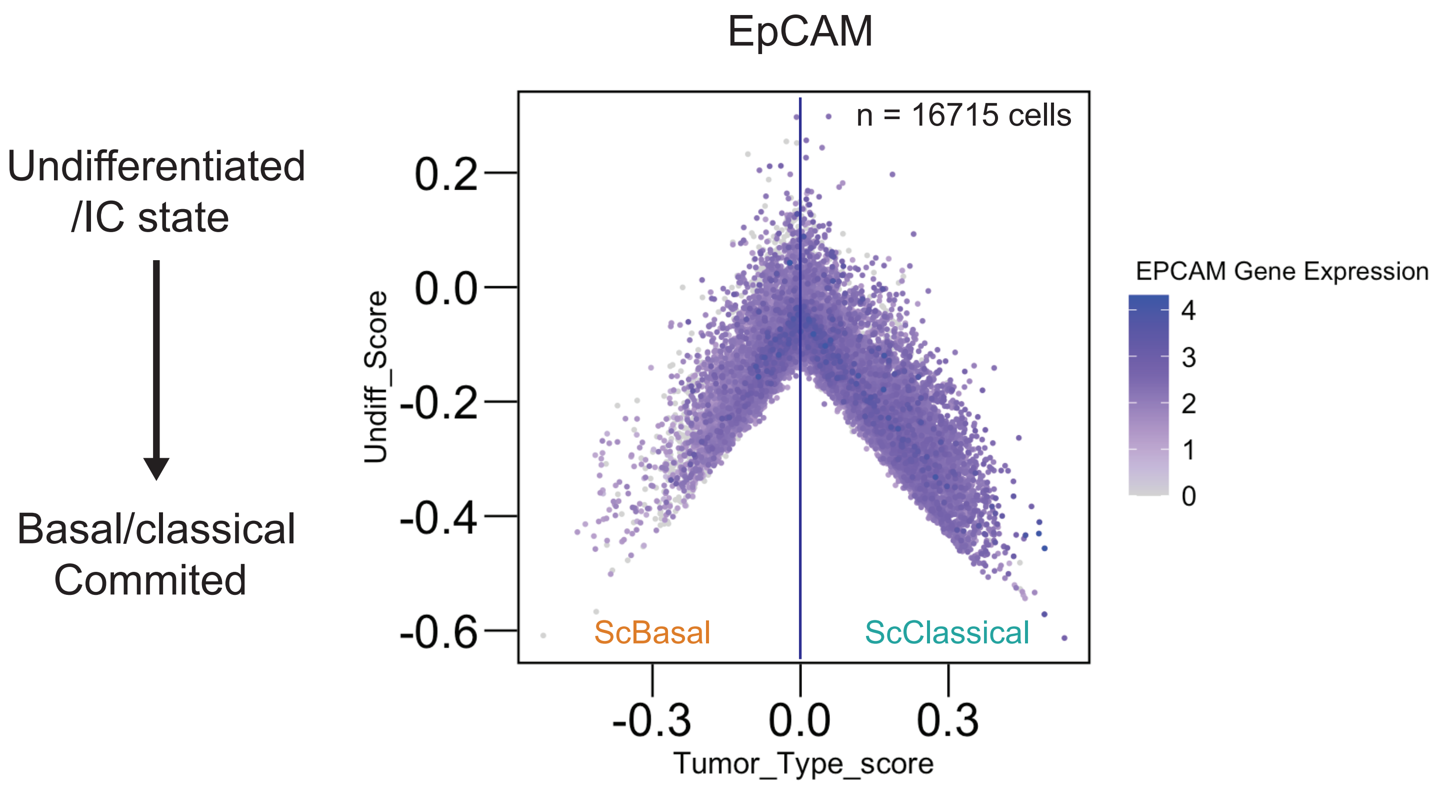

Wishbone graph showing a wide expression of EpCAM in both classical and basal scRNAseq samples.
